# Resonant-mode engineering for additive reflective structural colors with high brightness and high color purity

**DOI:** 10.1038/s41598-024-64176-4

**Published:** 2024-06-13

**Authors:** Hojae Kwak, Incheol Jung, Dohyun Kim, Seongcheol Ju, Soyoung Choi, Cheolhun Kang, Hyeonwoo Kim, Hyoung Won Baac, Jong G. Ok, Kyu-Tae Lee

**Affiliations:** 1 Department of Physics, Inha University, Incheon, 22111 Republic of Korea; 2 Department of Electrical and Computer Engineering, Sungkyunkwan University, Suwon, 16419 Republic of Korea; 3 Department of Mechanical and Automotive Engineering, Seoul National University of Science and Technology, Seoul, 01811 Republic of Korea

**Keywords:** Color filter, Multilayer, Fabry–Perot cavity, Higher-order resonance, Optics and photonics, Optical physics

## Abstract

We present quad-layered reflective structural color filters generating vivid additive primary colors by controlling a mode number in a Fabry–Perot (FP) cavity and an anti-reflective (AR) coating layer, thus accomplishing high spectral contrast which is highly demanded in creating sharp colors. The reflection brightness of fabricated structural color filters is over 78% and a color gamut is comparable to the standard color gamut (sRGB). Higher-order resonant modes are exploited yielding a narrow passband with strong suppression of the reflection at shorter and longer wavelength ranges for a green color, while red and blue colors are produced by employing fundamental resonant modes. Besides, the structural color filters maintain both high brightness and high color purity at oblique incidence angles up to 40° due to a small angle of refraction by a cavity medium with high refractive index. Moreover, a large-scale fabrication is enabled owing to the simplicity of a device structure, where thin film deposition is used. The scheme presented in this work may open the door to a number of applications, such as reflective displays, imaging devices, colored photovoltaics, and decorations.

## Introduction

Color filters are widely used over various areas, such as liquid crystal displays (LCDs), white organic light-emitting diode displays (WOLEDs), image sensors, and anti-counterfeiting devices^1–4^. However, since the existing color implementation is strongly dependent on the absorption of incident light with specific bands of wavelengths, conventional color filters based on dyes or pigments exhibit poor colors ascribing to their deficiencies in brightness, purity, and stability. To address the aforementioned issues, an enormous amount of effort has been devoted to finding the alternative of the conventional color filters, and structural color filters generating colors via light-matter interactions in which a narrow optical band can be achieved with relatively trivial losses leading to simultaneous achievement of both high brightness and high color purity have gained increasing attention. Numerous approaches based on multi-layered thin films^5–10^, guided-mode resonators^11–13^, plasmonic nanostructures^14–20^, Mie resonators^21–25^, and metasurfaces^26–31^ have been experimentally demonstrated, all of which present their superior color performances over the conventional color filters. Particularly, the multi-layered thin film structures are advantageous in that they can be created by a simple deposition process without a series of complicated and expensive lithographic techniques, thus offering the distinct potential for large-scale applications. While extensive research efforts have brought remarkable improvements in the brightness, purity, and stability, much attention has been devoted to the development of transmission-type additive structural color filters. Reflection-type additive structural color filters have remained unexploited although they play central roles in a variety of applications, such as electro phoretic displays (EPDs), outdoor signages, imaging devices, and automotive coatings. Although several approaches toward the reflective RGB color generation have been demonstrated, they rely on complicated fabrication techniques, and they present low brightness and poor color purity, thus imposing practical limitations on many potential applications. It is accordingly highly demanded to explore novel structures that are capable of generating additive colors in reflection with high brightness and high color purity.

In this work, we demonstrate engineering a resonant mode in a quad-layered structure consisting of a lossy metal sandwiched by two dielectric layers on a reflective mirror for the structural color filters creating additive primary colors of red (R), green (G), and blue (B) in reflection with high brightness and high color purity. An order of the resonance in the quad-layered structures is well controlled to achieve greatly improved optical properties, where higher-order resonances are employed for the G color whereas fundamental resonances are used for the B and R colors. All three RGB filters show high reflectance over 78% with the improved color purity exhibiting little discrepancy from the standard RGB color space. In addition, high index of refraction of a cavity medium allows the reflective colors to be nearly invariant when the light is incident at large angles of incidence up to 40˚. The simple design approach is expected to find a diversity of applications, such as reflective display technologies, colored solar cells, decorations, imaging devices, and surface coatings for automobiles.

## Results and discussion

**Figure 1:**
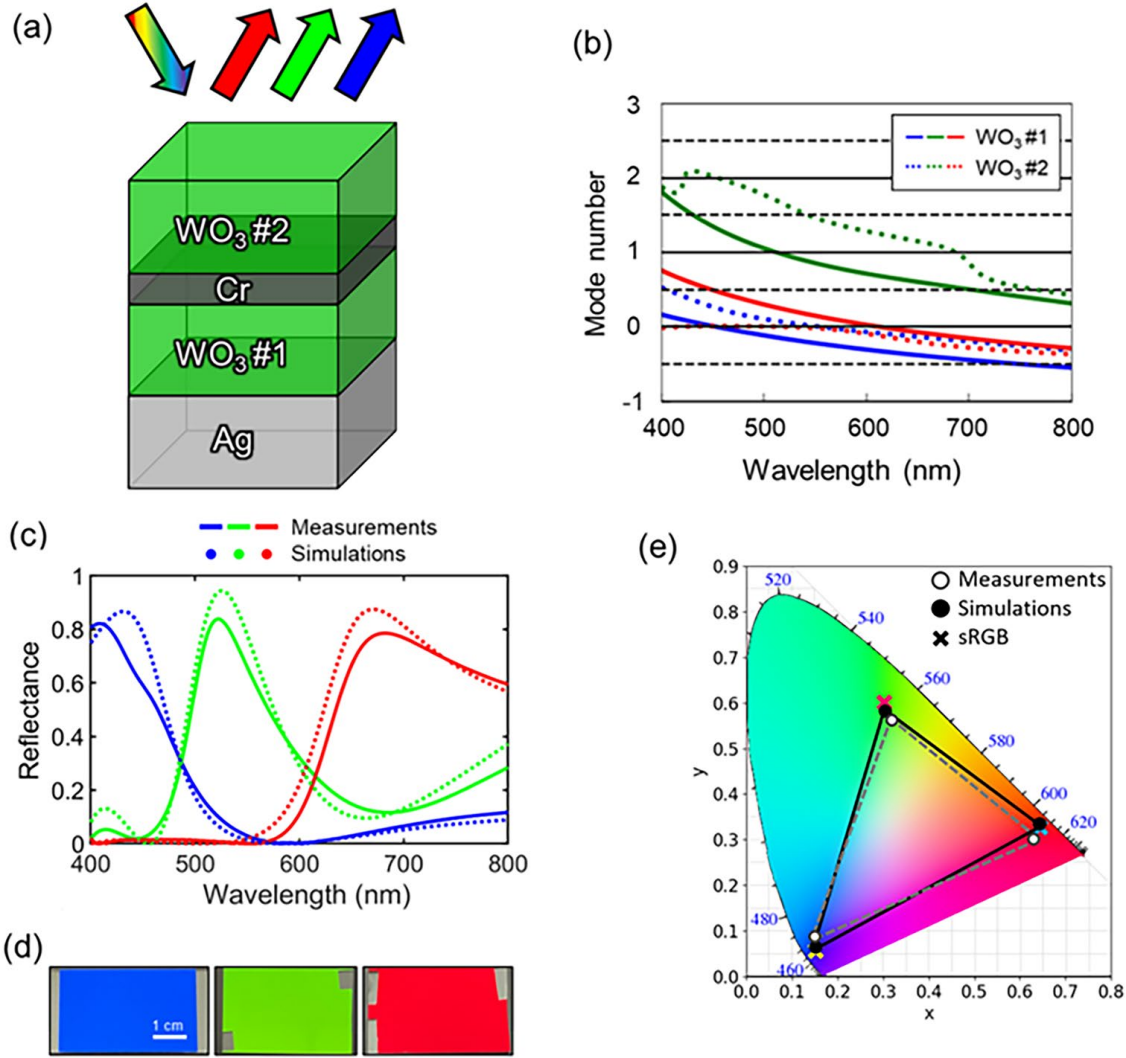
(**a**) Schematic diagram of proposed reflective RGB structural color filters. (**b**) Calculated resonant mode number, which is the net phase shift divided by 2π, in the WO_3_ #1 FP cavity and the WO_3_ #2 AR layer for the RGB. (**c**) Measured and simulated reflectance spectra of the reflective RGB structural color filters. (**d**) Optical images of the fabricated RGB color filter samples. (**e**) Color coordinates calculated from both the measured and simulated reflectance spectra along with the standard RGB color space (sRGB).

Figure 1a shows a schematic view of the proposed reflective RGB structural color filters which consist of the quad-layered thin films with a configuration of dielectric-metal-dielectric-metal (DMDM) on a glass substrate. Silver (Ag) is selected as a bottom layer whose thickness is optically thick providing high reflectivity. Tungsten trioxide (WO_3_) is chosen owing to negligible absorptions and high refractive index in the visible wavelength regime for the dielectric layers that comprise a Fabry–Perot (FP) cavity (WO_3_ #1) and an anti-reflective (AR) coating (WO_3_ #2). The lossy metal, chromium (Cr), is inserted between the two dielectric layers where constructive interference between light waves reflected from a surface of Cr and those reflected from the cavity occurs yielding a peak in the reflection^32,33^. Despite the high reflectance of a bulk Ag that is used at the bottom of the structure contributing to narrowing the full width at half maximum (FWHM), the high absorption of Cr leads to the low reflection and hence a broad reflectance peak thus causing the color performance to be significantly degraded. For the purpose of improving the color purity, the AR layer is well designed which suppresses the reflection of complementary colors (e.g., yellow for the B color filter). The resonant mode analysis which reveals the mode number of the FP resonance in each cavity medium, which is the net phase shifts divided by $2\pi$, where the net phase shift is the sum of reflectance phase shifts which occur at a top or a bottom interface of the cavity layer and a round-trip phase shift accumulated during the propagation inside the cavity medium is displayed in Fig. 1b. Black solid and dashed horizontal lines indicate conditions for constructive and destructive interferences, and colored solid and dashed lines stand for the resonant mode number in the WO_3_ #1 (FP cavities) and the WO_3_ #2 (AR) coating layers, respectively. As can be seen from the figure, the resonance is excited in the WO_3_ #1 FP cavity layer at wavelengths of 600 nm, 520 nm, and 450 nm for the RGB filters, respectively. Due to the peak transition effect, the reflectance peaks are formed adjacent to the resonant wavelengths. Meanwhile for the WO_3_ #2 AR layer, the destructive interference condition is satisfied at the wavelengths of 550 nm for the B filter, 450 nm and 700 nm for the G filter, and the wavelengths ranging from 400 to 550 nm for the R filter, respectively. Noticeably, an effective range of each AR coating well covers the wavelength regime of the complementary colors such that the AR effect can remarkably enhance the color purity. The manipulation of the number of the resonant mode in the FP cavity and the AR layer allows all the three RGB filters to attain high color purity as well as high brightness. Figure 1c shows measured (solid) and simulated (dotted) reflection spectra at normal incidence, presenting little discrepancy which is attributed to defects occurred during the deposition and errors in evaluating optical constants and film thicknesses. As shown in the figure, the measured spectra have their maxima of 78.6%, 83.6%, and 82.2% at the wavelengths of 683 nm, 522 nm, and 409 nm, respectively, and the corresponding values of 87.5%, 94.4%, and 86.7% at the wavelengths of 671 nm, 525 nm, and 431 nm, respectively, for the RGB colored samples. Optical photographs of fabricated RGB samples (2.5 cm $\times$ 5.0 cm) are shown in Fig. 1d displaying distinct RGB colors in reflection. CIE *xy* color coordinates are estimated from the measured and simulated spectral curves of reflectance, displayed in the CIE 1931 chromaticity diagram as described in Fig. 1e. The color coordinates are (0.63, 0.30), (0.32, 0.56), and (0.15, 0.08) from the measured data (grey), while (0.64, 0.33), (0.30, 0.57), and (0.15, 0.06) are obtained from the simulated results (black) for the RGB color filters. It is noted that the color coordinates of the proposed filters are close to the sRGB standard values which are (0.64, 0.33), (0.30, 0.60), and (0.15, 0.06). It is also noteworthy that other noble metals like Al or Au would replace Ag for the bottom reflector, but both the brightness and the color purity are significantly degraded by replacing the Ag bottom reflector with the lossy metals like Cr or Ti which are provided in Supporting Information (Figs. S1 and S2). A table for a comprehensive comparison with prior works based on the FP and surface plasmon polariton (SPP) is provided in the Supporting Information (Table S1).

Thicknesses of each layer in the quad-layered RGB color filters are optimized by employing a numerical optimization method based on Broyden–Fletcher–Goldfarb–Shanno (BFGS) algorithm^34^, which are provided in Table 1. All the calculations for the analysis and simulations are performed by Abeles transfer matrix method as well as a commercially available software (Essential Macleod), and the optical constants of the materials that are obtained by using a spectroscopic ellipsometer (VASE, J. A. Woollam) are illustrated in Fig. S3(a) and (b). The device fabrication is carried out by using an electron beam evaporator, and the spectral responses of the fabricated devices are measured by using a spectrophotometer (Cary 500, Varian).

**Table 1:** Materials and thicknesses of the constituent layers of the proposed reflective RGB structural color filters.

Material	Thickness (nm)		
	Blue	Green	Red
WO_3_	76	134	41
Cr	18	13	21
WO_3_	68	222	130
Ag	100	100	100

**Figure 2:**
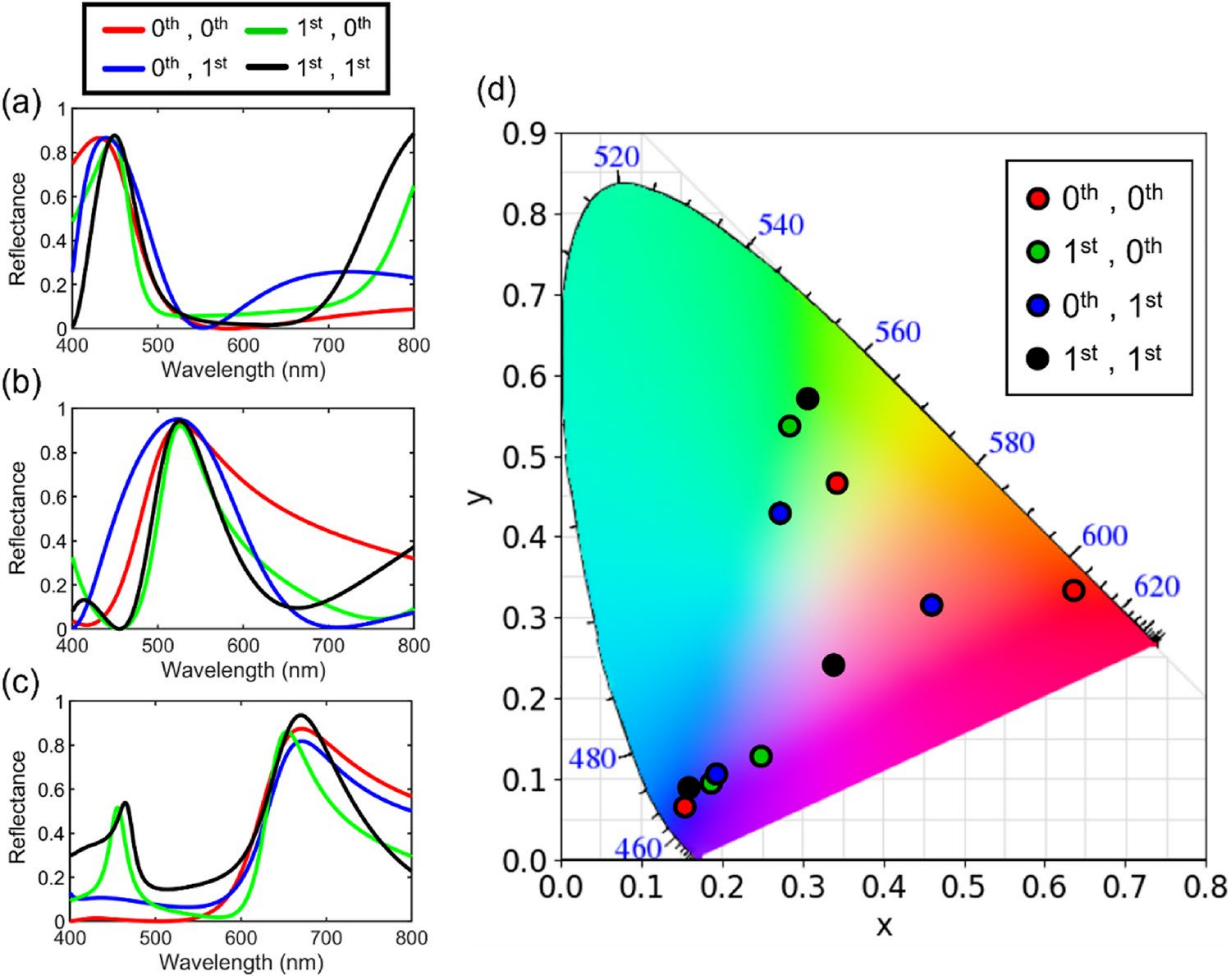
Calculated spectral curves of reflectance for the (**a**) B, (**b**) G, and (**c**) R color filters with the different resonance orders in the WO_3_ #1 and #2 layers, and (**d**) the corresponding color coordinates described on the CIE 1931 chromaticity diagram.

To create pure colors, it is apparent that the passband of the filter should be as narrow as possible while the spectral contrast which is the difference between the efficiencies of the passband and the stopband should be high. To achieve both the narrow passband with the high spectral contrast, an order of the resonant mode is exploited when designing the cavity since both the bandwidth and the brightness are largely affected by the mode number of the resonance. We note that the spectrum outside the visible regime of the human eye also functions as a natural stopband thus the narrow passband is not necessary in creating pure colors when it comes to B or R both of which are at the extremes of the visible spectrum. Therefore, the optimal combinations of the resonance modes for the RGB colors are (0th, 0th), (1st, 1st), and (0th, 0th), respectively, with the notation as follows: (${m}_{\#1}$, ${m}_{\#2}$), where ${m}_{\#1}$ and ${m}_{\#2}$ denote the mode number in the WO_3_ #1 and #2 layers. As an advanced analysis on the mode combinations, we also explore the remaining combinations of the resonance modes, the results of which are illustrated in Fig. 2. We investigate on four different combinations of (0th, 0th), (0th, 1st), (1st, 0th), and (1st, 1st) orders for the each WO_3_ #1 and #2 layers by simulating the spectral responses of the designs and then evaluating the color coordinates from the spectral data. The reflectance spectra of (0th, 0th), (0th, 1st), (1st, 0th), and (1st, 1st) are visualized as red, blue, green, and black solid lines in Fig. 2a–c, and the color coordinates are depicted as circles with the corresponding colors in Fig. 2d. For the B filter, the bandwidth of the passband does not have a significant impact on the color performance but only the degree of suppression. The mode combination of (0th, 0th) serves the strongest suppression for the wavelengths longer than ~ 500 nm, resulting in the highest color purity. The G color is in the middle of the visible regime thus requiring the filter to have its passband to be narrow and to have two stopbands in the shorter and longer wavelengths. The higher-order resonance in the WO_3_ #1 layer leads to the narrow passband and that in the #2 layer incurs the higher mode in the shorter wavelength such that two stopbands are provided by a single layer. The trend is also observed in Fig. 2b and d, in which (1st, 1st) set attains the highest color purity and the exceedingly high brightness of 94.4%. In case of the R filter, as mentioned above, avoiding the undesired higher-order mode that hinders creating the pure R color is the top priority, so the fundamental mode is used in the WO_3_ #1 layer. Also, since the stopband of the AR coating is inversely proportional to the center wavelength, it is more difficult to suppress sufficient range of wavelengths for the R filter which requires suppression in the B and G regions, thus identically to that in the B filter the fundamental mode is again the best option for the #2 layer.

For the further analysis, optical admittance diagrams by which the revolution of the optical admittance of the multi-layered structure is identified in accordance with the film thickness and the refractive index of the constituent layers of the entire structure are studied. The optical admittance, the ratio of the electric field to the magnetic field, is a dominant factor that determines the optical behavior against incoming light therefore being one of the most useful tools to investigate the optical responses in the multi-layered structures. The value of a single material can be calculated by taking a square root of the ratio of the permittivity to the permeability ($Y=\sqrt{\varepsilon /\mu }$; $Y$, $\varepsilon$, and $\mu$ denote optical admittance, permittivity, and permeability, respectively), and since the permeability approximates to one for non-magnetic materials, it can be regarded to be equivalent to the refractive index of the material which equals to the square root of permittivity ($Y\approx \sqrt{\varepsilon }=n$; *n* stands for refractive index). In the multi-layered structures, the optical admittance evolution can be characterized by the admittance loci which can be calculated by applying the transfer matrix method, where a termination point of the loci represents a surface admittance which determines the optical behavior of the multi-layered structure^35^. Assuming that the surface admittance is $Y$ and the optical admittance of the surrounding medium is ${Y}_{0}$, the reflectance can be calculated as follows:

1 $$R={\left|\frac{{Y}_{0}-Y}{{Y}_{0}-Y}\right|}^{2}$$

**Figure 3:**
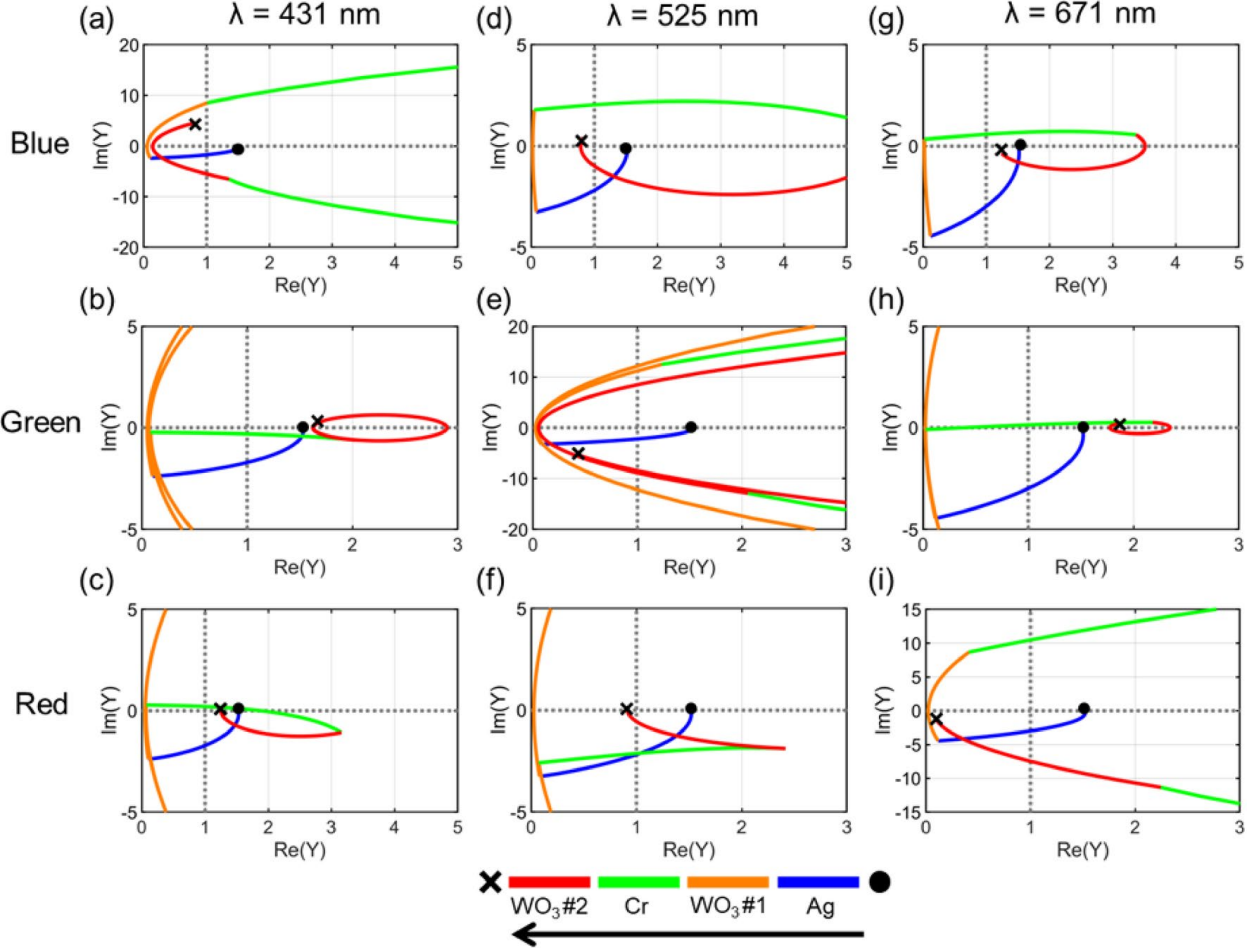
Admittance diagrams of the B (**a**, **d**, **g**), G (**b**, **e**, **h**), and R (**c**, **f**, **i**) colored structures at the wavelengths of 431 nm, 525 nm, 671 nm where a maximum reflectance occurs. Black circles represent starting points of the admittance and black x marks describe the termination points of the admittance. The admittance loci are determined by the thickness and the refractive index of the constituent layers of the structure, where Ag, WO_3_ #1, Cr, and WO_3_ #2 are denoted by blue, orange, green, and red colors.

Figure 3 shows the optical admittance diagrams of the proposed RGB reflective structural color filters at specific wavelengths of 431 nm, 525 nm, and 671 nm at which the RGB filters show their maximum value of the reflectance. A black circle located at (1.52, 0) is the admittance starting point which equals to the optical admittance of a glass substrate; the blue (Ag), orange (WO_3_ #1), green (Cr), and red (WO_3_ #2) solid lines indicate the evolution of the optical admittance inside the constituent layers; and the black × mark depicts the admittance termination point which identifies the surface admittance of the filter. As the reflectance of the multi-layered structures is proportional to the absolute value of the square of the distance from the surface admittance to the admittance of the surrounding medium, the further the admittance termination point of the filter from the admittance of air, the stronger reflection is anticipated. The admittance termination points are (0.81, 4.14), (0.81, 0.21), and (1.24, − 0.21) for the B filter, those are (1.67, 0.34), (0.43, − 5.00), and (1.87, 0.14) for the G filter, and those are (1.24, 0.08), (0.91, 0.88), (0.11, − 1.25) for the R filter at the wavelengths of 431 nm, 525 nm, and 671 nm, respectively. In the all RGB filters, the admittance termination points are distant from the admittance of air, (1, 0), at the wavelengths of their maximum reflectance, whereas at the wavelengths included in the stopbands the two points are adjacent to each other, implying the existence of strong reflective suppression outside the passband. The results of the investigation are highly consistent to the spectral responses that are obtained from both the simulations and measurements, as illustrated in Fig. 1c.

**Figure 4:**
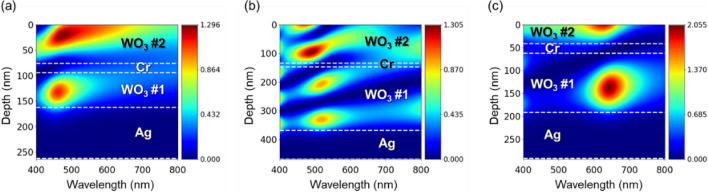
2D contour plots of the electric filed distribution as functions of the wavelength and a depth into the structural color filters for the (**a**) B, (**b**) G and (**c**) R filters.

Electric field intensity profiles as a function of the wavelength and a depth into the structures are illustrated in Fig. 4. The depth is zero at the surface of the filter then increases as penetrating into the structure. In Fig. 4a, the electric field is concentrated at the wavelength of 450 nm in the WO_3_ #1 layer, and over a wide wavelength range from 430 to 600 nm in the WO_3_ #2 layer. The field confinement in the WO_3_ #1 layer indicates the existence of the FP resonance resulting in a reflective peak at the resonant wavelength owing to the Cr absorptive mirror located at the illumination side. On the other hand, as the WO_3_ #2 layer provides the AR effect, the electric field is focused over the range of the wavelengths at which the AR is effective. However, since the Air-WO_3_ interface provides too weak reflection as compared to that occurs on the other side of the WO_3_ #2 layer, the field confinement is extended to the surface, as depicted in Fig. 4a. The effective region of the AR effect can be roughly estimated by the confined regime of the electric field at the surface of the structure, showing a good agreement with the spectrum in Fig. 1c in which a strong suppression of the reflectance over the same wavelength range is observed. Similarly, the concentrated electric field exists in the WO_3_ #1 and #2 layers in Fig. 4b, where they respectively attribute to the FP resonance and the AR effect. However, each layer has two focusing points at the wavelength of 520 nm in the WO_3_ #1 and at the wavelength of 460 nm in the WO_3_ #2, indicating that the higher-order resonant modes are excited as described in the previous section. In addition, as intended for the WO_3_ #2 layer of the G filter to provide the AR effect at two different wavelengths in order for enhancing the spectral contrast, a weak but apparent field confinement is also identified at the wavelength of 650 nm in the WO_3_ #2 layer, which can validate the existence of additional AR effect providing a spectral suppression at the longer wavelength region. Since the resonant mode occurring at the longer wavelength regime is fundamental, only one concentration point occurs at the wavelength of 650 nm. When it comes to the R filter, both the AR effect and the FP resonance have the fundamental mode in order for providing a strong suppression over a wide range of wavelengths from 400 to 550 nm, so the electric field intensity is confined in a single point at the wavelengths of 650 nm and 630 nm in the WO_3_ #1 and WO_3_ #2 layers, respectively. Although the AR effect appearing at the wavelength of 630 nm seems to be too adjacent to the resonant wavelength of the FP cavity, since the reflectance peak is not formed at the exact resonant wavelength but is slightly red-shifted due to a slightly dislocated condition of constructive interference attributing to the optical response of the top bi-layer of the effective mirror, it highly contributes to the improvement of the color purity without sacrificing the brightness by providing a strong suppression at the wavelengths around 600 nm which are responsible for generating an orange color.

**Figure 5:**
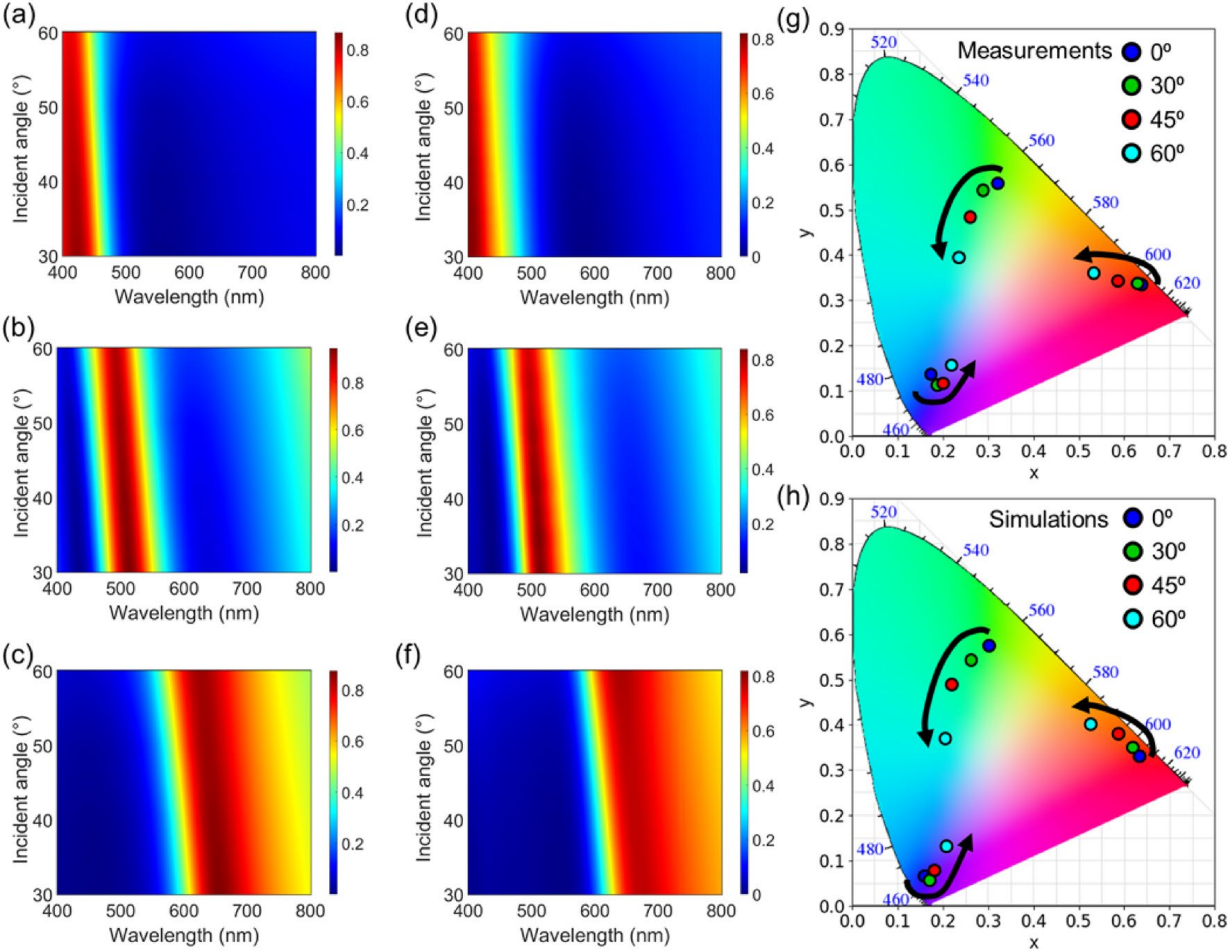
Measured (**a**–**c**) and simulated (**d**–**f**) angle-resolved reflectance spectra of the proposed structural color filters. (**g**) Measured and (**h**) simulated color coordinate changes with increasing the incident angles under the mean polarization presented on CIE 1931 chromaticity diagram.

Lastly, the evolution of the optical response of the proposed filters when the light is incident at oblique angles up to 60° is examined. The optical resonance is sensitive to the incident angle, but the sensitivity depends on the refractive index of the cavity medium. The change in the resonant wavelength (${\lambda }_{r}$) with the incident angle (${\theta }_{1}$) is inversely proportional to the refractive index of the cavity medium ($\frac{{\Delta\uplambda }_{\text{r}}}{{\Delta{\theta}}_{1}}=\frac{2 \text{d } \cdot {\sin}{\uptheta}_{1} \cdot \cos{\uptheta}_{1}}{\sqrt{{\text{n}}^{2} \cdot {{\sin}}^{2}{\uptheta}_{1}}}$), implying that the angular performance can be improved by employing the high-index medium for the cavity layer^36^. Figure 5a–c shows measured angle-resolved reflectance spectra showing good agreement with simulated profiles as exhibited in Fig. 5d–f. The corresponding color coordinates are described in Fig. 5g and h. The ellipsometer is used to characterize the angular performance of the fabricated RGB color filters for the incident angles from 30° to 60°, and the angle-resolved spectral simulations are carried out by using the transfer matrix method. Although a blue-shift in the reflectance is observed according to the increment of the incident angle, owing to the high refractive index of the WO_3_ that is used as the cavity medium, a spectral change is trivial exhibiting nearly constant spectra up to the incident angle of 40° under the illumination of unpolarized light. For the B filter, the peak wavelength in the reflectance varies from 410 nm (0°) to 405 nm (30°), 400 nm (45°), and then 395 nm (60°) in the measurement, from 431 nm (0°) to 423 nm (30°), 415 nm (45°), and then 410 nm (60°) in the simulation, as illustrated in Fig. 5a and d, respectively. Since the B color is at the shortest edge of the visible spectrum, the blue-shift little affects the purity of the generated colors thus the filter is quite robust to the change of the incident angle, as displayed in Fig. 5g and h, in which the color coordinates are illustrated as (0.175, 0.147), (0.185, 0.114), (0.196, 0.120), and (0.215, 0.156) for 0°, 30°, 45°, and 60° in the measurement, and (0.156, 0.066), (0.164, 0.058), (0.179, 0.072), and (0.213, 0.142) for 0°, 30°, 45°, and 60° in the simulation, respectively. For the G filter, on the other hand, since it utilizes the higher-order modes for both the FP cavity and the AR layer, the angle of incidence inflicts much stronger implication on the blue-shift phenomenon thereby deteriorating the angular color performance. It is noticeable by the Fig. 5b and e, in which a steeper change of the reflective peaks according to the incident angle is observed, where the peak locations are 520 nm (0°) to 515 nm (30°), 505 nm (45°), and 495 nm (60°) in the measurement, and 525 nm (0°) to 514 nm (30°), 503 nm (45°), and 492 nm (60°) in the simulation, respectively. Accordingly, the generated colors also are highly dependent on the angle of incidence, where the specific coordinates are evaluated as (0.320, 0.569), (0.292, 0.545), (0.259, 0.489), and (0.231, 0.396) for 0°, 30°, 45°, and 60° in the measurement, and (0.304, 0.574), (0.261, 0.550), (0.224, 0.479), and (0.205, 0.365) for 0°, 30°, 45°, and 60° in the simulation, as described in Fig. 5g and h, respectively. On yet another hand, despite the use of the fundamental modes in both the WO_3_ layers in the R filter, since the shifted peak intrudes into the G area as it blue-shifts therefore engendering a strong spectral noise, the colors vastly deteriorates as the incident angle increases. The variation of the peak wavelengths is from 680 nm (0°) to 675 nm (30°), 660 nm (45°), and then 640 nm (60°) in the measurement, from 671 nm (0°) to 656 nm (30°), 642 nm (45°), and then 626 nm (60°) in the simulation, as depicted in Fig. 5c and f, respectively, whereas the color coordinate change is from (0.645, 0.335), (0.632, 0.339), (0.597, 0.348), and then (0.530, 0.358) for 0°, 30°, 45°, and 60° in the measurement, and from (0.638, 0.333), (0.621, 0.354), (0.587, 0.379), and then (0.522, 0.400) for 0°, 30°, 45°, and 60° in the simulation, as visualized in Fig. 5g and h, respectively. Noticeably, the coordinates move into the orange region at high incident angles due to the blue-shift phenomenon. In addition, we note that the higher reflectance over the whole wavelengths attributing to the higher angles of the incident lights causes a strong spectral noise thereby degrading the color saturation, as commonly appears in all three filters as illustrated in Fig. 5g and h. Angular performances and the corresponding color coordinates for p- and s-polarizations are provided in Figs. S4 and S5.

## Conclusion

In conclusion, we have demonstrated the improved brightness and color purity of the quad-layered reflective structural color filters by controlling the order of the resonant mode in the FP cavity and the AR layer. The presented devices attain high brightness over 78% and generates pure reflective RGB colors whose color gamut is analogous to that of the sRGB. Moreover, rigorous analyses on the device structures by exploiting the net phase analysis, optical admittance loci, and electric field intensity distributions are conducted to provide a better understanding. Furthermore, the angular performance of the color filters is investigated, exhibiting that the angle-tolerant characteristic is maintained up to 40° of the incident angle owing to the reduced refraction angle into the cavity by high refractive index of the cavity medium. Additionally, only the thin film deposition is required for the device fabrication, bolstering the readiness of mass-production of the large-scale devices. The demonstrated strategy may pave the way to various applications, such as reflective displays, imaging devices, optical sensors, color solar panels, and decorations.

### Supplementary Information

Supplementary Information.

## Data Availability

The datasets used and/or analyzed during the current study available from the corresponding author on reasonable request.

## References

[1] Sabnis RW (1999). Color filter technology for liquid crystal displays. Displays.

[2] Chang CH, Cheng HC, Lu YJ, Tien KC, Lin H-W, Lin CL, Yang CJ, Wu CC (2010). Enhancing color gamut of white OLED displays by using microcavity green pixels. Org. Electron..

[3] Yokogawa S, Burgos SP, Atwater HA (2012). Plasmonic color filters for CMOS image sensor applications. Nano Lett..

[4] Xuan Z, Li J, Liu Q, Yi F, Wang S, Lu W (2021). Artificial structural colors and applications. Innovation.

[5] Yang C, Shen W, Zhang Y, Li K, Fang X, Zhang X, Liu X (2015). Compact multilayer film structure for angle insensitive color filtering. Sci. Rep..

[6] Lee K-T, Seo S, Lee JY, Guo LJ (2014). Strong resonance effect in a lossy medium-based optical cavity for angle robust spectrum filters. Adv. Mater..

[7] Ghobadi A, Hajian H, Soydan MC, Butun B, Ozbay E (2019). Lithography-free planar band-pass reflective color filter using a series connection of cavities. Sci. Rep..

[8] Yoo YJ, Lim JH, Lee GJ, Jang KI, Song YM (2017). Ultra-thin films with highly absorbent porous media fine-tunable for coloration and enhanced color purity. Nanoscale.

[9] Li Z, Butun S, Aydin K (2015). Large-area, lithography-free super absorbers and color filters at visible frequencies using ultrathin metallic films. ACS Photonics.

[10] Kim D, Kim H, Jung I, Kim TY, Kwak H, Jung JH, Hwangbo CK, Park HJ, Lee K-T (2022). Manipulation of resonance orders and absorbing materials for structural colors in transmission with improved color purity. Opt. Express.

[11] Kaplan AF, Xu T, Guo LJ (2011). High efficiency resonance-based spectrum filters with tunable transmission bandwidth fabricated using nanoimprint lithography. Appl. Phys. Lett..

[12] Uddin MJ, Khaleque T, Magnusson R (2014). Guided-mode resonant polarization-controlled tunable color filters. Opt. Express.

[13] Shrestha VR, Lee S-S, Kim E-S, Choi D-Y (2015). Polarization-tuned dynamic color filters incorporating a dielectric-loaded aluminum nanowire array. Sci. Rep..

[14] Xu T, Wu YK, Luo X, Guo LJ (2010). Plasmonic nanoresonators for high-resolution colour filtering and spectral imaging. Nat. Commun..

[15] Kumar K, Duan H, Hegde RS, Koh SCW, Wei JN, Yang JKW (2012). Printing colour at the optical diffraction limit. Nat. Nanotechnol..

[16] Roberts AS, Pors A, Albrektsen O, Bozhevolnyi SI (2014). Subwavelength plasmonic color printing protected for ambient use. Nano Lett..

[17] Li Z, Clark AW, Cooper JM (2016). Dual color plasmonic pixels create a polarization controlled nano color palette. ACS Nano.

[18] Yang C, Shen W, Zhou J, Fang X, Zhao D, Zhang X, Ji C, Fang B, Zhang Y, Liu X, Guo LJ (2016). Angle robust reflection/transmission plasmonic filters using ultrathin metal patch array. Adv. Opt. Mater..

[19] Shrestha VR, Lee S-S, Kim E-S, Choi DY (2014). Aluminum plasmonics based highly transmissive polarization-independent subtractive color filters exploiting a nanopatch array. Nano Lett..

[20] Zeng B, Gao Y, Bartoli FJ (2013). Ultrathin nanostructured metals for highly transmissive plasmonic subtractive color filters. Sci. Rep..

[21] Tsai MC, Tsai TL, Lin CT, Chung RJ, Sheu HS, Chiu HT, Lee CY (2008). Tailor made Mie scattering color filters made by size-tunable titanium dioxide particles. J. Phys. Chem. C.

[22] Wood T, Naffouti M, Berthelot J, David T, Claude JB, Métayer L, Delobbe A, Favre L, Ronda A, Berbezier I, Bonod N, Abbarchi M (2017). All-dielectric color filters using SiGe-based Mie resonator arrays. ACS Photonics.

[23] Jang J, Badloe T, Yang Y, Lee T, Mun J, Rho J (2020). Spectral modulation through the hybridization of Mie-scatterers and quasi-guided mode resonances: Realizing full and gradients of structural color. ACS Nano.

[24] Alam A-M, Baek K, Son J, Pe Y-R, Kim DH, Choy J-H, Hyun JK (2017). Generating color from polydisperse, near micron-sized TiO_2_ particles. ACS Appl. Mater. Interfaces.

[25] Jang J, Badloe T, Sim YC, Yang Y, Mun J, Lee T, Cho Y-H, Rho J (2020). Full and gradient structural colouration by lattice amplified gallium nitride Mie-resonators. Nanoscale.

[26] Duan X, Kamin S, Liu N (2017). Dynamic plasmonic colour display. Nat. Commun..

[27] Yang W, Xiao S, Song Q, Liu Y, Wu Y, Wang S, Yu J, Han J, Tsai DP (2020). All-dielectric metasurface for high-performance structural color. Nat. Commun..

[28] Park C-S, Shrestha VR, Yue W, Gao S, Lee S-S, Kim E-S, Choi D-Y (2017). Structural color filters enabled by a dielectric metasurface incorporating hydrogenated amorphous silicon nanodisks. Sci. Rep..

[29] Galinski H, Favraud G, Dong H, Gongora JST, Favaro G, Döbeli M, Spolenak R, Fratalocchi A, Capasso F (2017). Scalable, ultra-resistant structural colors based on network metamaterials. Light Sci. Appl..

[30] Sun S, Zhou Z, Zhang C, Gao Y, Duan Z, Xiao S, Song Q (2017). All-dielectric full-color printing with TiO_2_ metasurfaces. ACS Nano.

[31] Yoon G, So S, Kim M, Mun J, Ma R, Rho J (2017). Electrically tunable metasurface perfect absorber for infrared frequencies. Nano Converg..

[32] Rana AS, Zubair M, Anwar MS, Saleem M, Mehmood MQ (2020). Engineering the absorption spectra of thin film multilayer absorbers for enhanced color purity in CMY color filters. Opt. Mater. Express.

[33] Jung I, Kim H, Oh S, Kwak H, Ju S, Kim M, Jung JH, Baac HW, Ok JG, Lee K-T (2023). Understanding a spectral response in a metal–dielectric–metal cavity structure: The role of constituent metals. Opt. Laser Technol..

[34] Byrd RH, Lu P, Nocedal J, Zhu C (1995). A limited memory algorithm for bound constrained optimization. SIAM J. Sci. Comput..

[35] Ohta K, Ishida H (1990). Matrix formalism for calculation of electric field intensity of light in stratified multilayered films. Appl. Opt..

[36] Ji C, Lee K-T, Xu T, Zhou J, Park HJ, Guo LJ (2017). Engineering light at the nanoscale: Structural color filters and broadband perfect absorbers. Adv. Opt. Mater..

