# Supplementary material for: Resonant-mode engineering for additive reflective structural colors with high brightness and high color purity
Source: Sci Rep. 2024 Jun 13;14:13694. doi: 10.1038/s41598-024-64176-4 (PMC11176299; doi:10.1038/s41598-024-64176-4)
Supplement: Supplementary file 1 — Supplementary Information. [file 41598_2024_64176_MOESM1_ESM.pdf]

Supporting Information

Resonant-mode engineering for additive reflective structural colors with high brightness and high color purity

Hojae Kwak [1,†], Incheol Jung [1,†], Dohyun Kim [1,†], Seongcheol Ju [1], Soyoung Choi [1], Cheolhun Kang [1], Hyeonwoo Kim [1], Hyoung Won Baac [2,*], Jong G. Ok [3,*], and Kyu-Tae Lee [1,*]

† These authors contributed equally.

* Corresponding author.

[1] Department of Physics, Inha University, Incheon 22111, Republic of Korea

[2] Department of Electrical and Computer Engineering, Sungkyunkwan University, Suwon, 16419, Republic of Korea

[3] Department of Mechanical and Automotive Engineering, Seoul National University of Science and Technology, Seoul, 01811, Republic of Korea

* Prof. H. W. Baac (hwbaac@skku.edu)

*Prof. J. G. Ok (jgok@seoultech.ac.kr)

*Prof. K.-T. Lee (ktlee@inha.ac.kr)

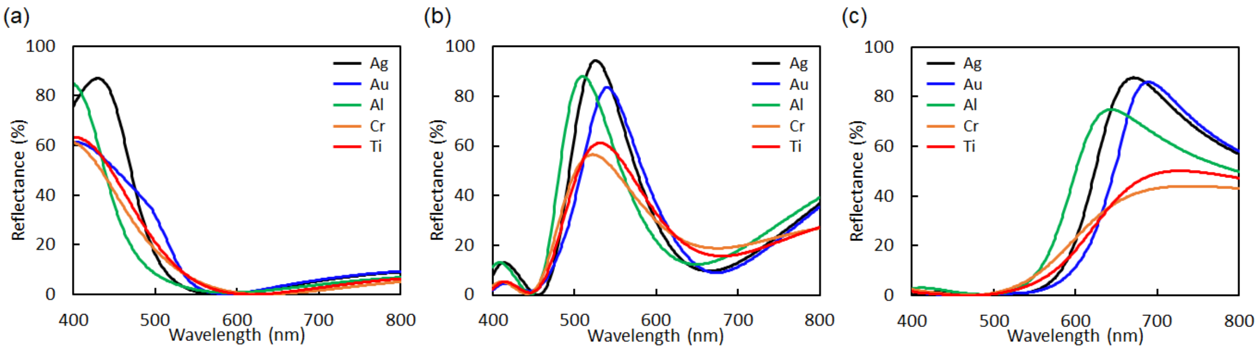

Figure S1. Reflectance spectra of the reflective RGB structural color filters with the different bottom reflector for (a) blue, and (b) green, and (c) red.

(a)

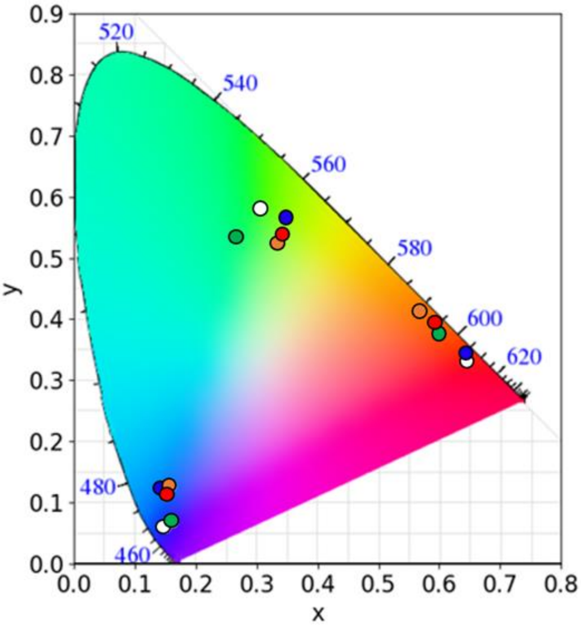

(b)

Bulk		(x, y)		
		Blue	Green	Red
○	Ag	(0.15, 0.06)	(0.30, 0.57)	(0.64, 0.33)
●	Au	(0.15, 0.12)	(0.35, 0.56)	(0.64, 0.34)
●	Al	(0.16, 0.07)	(0.27, 0.53)	(0.60, 0.37)
●	Cr	(0.16, 0.12)	(0.33, 0.52)	(0.57, 0.41)
●	Ti	(0.15, 0.11)	(0.34, 0.53)	(0.59, 0.39)

Figure S2. (a) Color coordinates calculated from the reflectance spectra shown in Figure S1 illustrated on the CIE 1931 chromaticity diagram, and (b) color spaces.

Table S1. A summary comparison of the peak efficiency in reflection and the color space of our work and prior works based on the Fabry-Perot and surface plasmon polariton.

Reflective RGB			Our work	[1]	[2]	[3]	[4]	[5]	[6]	[7]
Mechanism			Thin film interference					SPP: Surface Plasmon Polariton		
Structure			Quad-layer [DMDM]	Tri-layer [MDM]	Penta-layer [DMMDM]	Quad-layer [DMSM]	Penta-layer [SDMDM]			
			D: Dielectric, M: Metal, S: Semiconductor							
Peak Efficiency (%) Exp (Sim)	Red		78.6 (87.5)	91.4 (92.9)	70.1 (74.0)	56.8 (76.9)	70.0 (76.9)	17.2 (17.1)	64.2 (93.5)	65.8 (65.9)
	Green		83.6 (94.4)	88.1 (92.4)	61.2 (63.0)	45.5 (48.5)	61.8 (66.1)	12.0 (12.5)	45.3 (82.3)	73.3 (70.3)
	Blue		82.2 (86.7)	78.9 (91.0)	72.2 (73.0)	76.4 (83.9)	61.0 (66.3)	5.5 (5.5)	36.5 (62.8)	71.7 (70.8)
Color space (x, y)	Red	sRGB	(0.64, 0.33)							
		Exp	(0.63, 0.30)	(0.44, 0.43)	(0.62, 0.31)	(0.54, 0.32)	(0.52, 0.35)	(0.42, 0.38)	(0.43, 0.37)	(0.49, 0.31)
		Sim	(0.64, 0.33)	(0.47, 0.45)	(0.64, 0.33)	(0.54, 0.31)	(0.45, 0.29)	(0.48, 0.43)	(0.47, 0.32)	(0.59, 0.37)
	Green	sRGB	(0.30, 0.60)							
		Exp	(0.32, 0.56)	(0.30, 0.37)	(0.31, 0.59)	(0.28, 0.43)	(0.24, 0.55)	(0.38, 0.39)	(0.37, 0.43)	(0.28, 0.46)
		Sim	(0.30, 0.57)	(0.32, 0.38)	(0.30, 0.60)	(0.29, 0.44)	(0.30, 0.53)	(0.43, 0.47)	(0.42, 0.50)	(0.30, 0.52)
	Blue	sRGB	(0.15, 0.06)							
		Exp	(0.15, 0.08)	(0.19, 0.18)	(0.15, 0.06)	(0.18, 0.23)	(0.14, 0.21)	(0.25, 0.29)	(0.23, 0.26)	(0.19, 0.11)
		Sim	(0.15, 0.06)	(0.16, 0.15)	(0.15, 0.06)	(0.16, 0.17)	(0.16, 0.33)	(0.18, 0.33)	(0.16, 0.16)	(0.21, 0.09)

[1] Z. Yang, et al., "Reflective Color Filters and Monolithic Color Printing Based on Asymmetric Fabry–Perot Cavities Using Nickel as a Broadband Absorber," *Adv. Opt. Mater.* **4**, 1196 (2016).

[2] Z. Yang, et al., "Enhancing the Purity of Reflective Structural Colors with Ultrathin Bilayer Media as Effective Ideal Absorbers," *Adv. Opt. Mater.* **7**, 1900739 (2019).

[3] C. Yang, et al., "Compact Multilayer Film Structure for Angle Insensitive Color Filtering," *Sci. Rep.* **5**, 9285 (2015).

[4] A. Ghobadi, et al., "Lithography-Free Planar Band-Pass Reflective Color Filter Using A Series Connection of Cavities," *Sci. Rep.* **9**, 290 (2019).

[5] L. Wen, et al., "Multifunctional Silicon Optoelectronics Integrated with Plasmonic Scattering Color," *ACS Nano* **10**, 11076 (2016).

[6] H. Wang, et al., "Full Color Generation Using Silver Tandem Nanodisks," *ACS Nano* **11**, 4419 (2017).

[7] E. G. Melo, et al., "Bright and Vivid Diffractive–Plasmonic Reflective Filters for Color Generation," *ACS Appl. Nano Mater.* **3**, 1111 (2020).

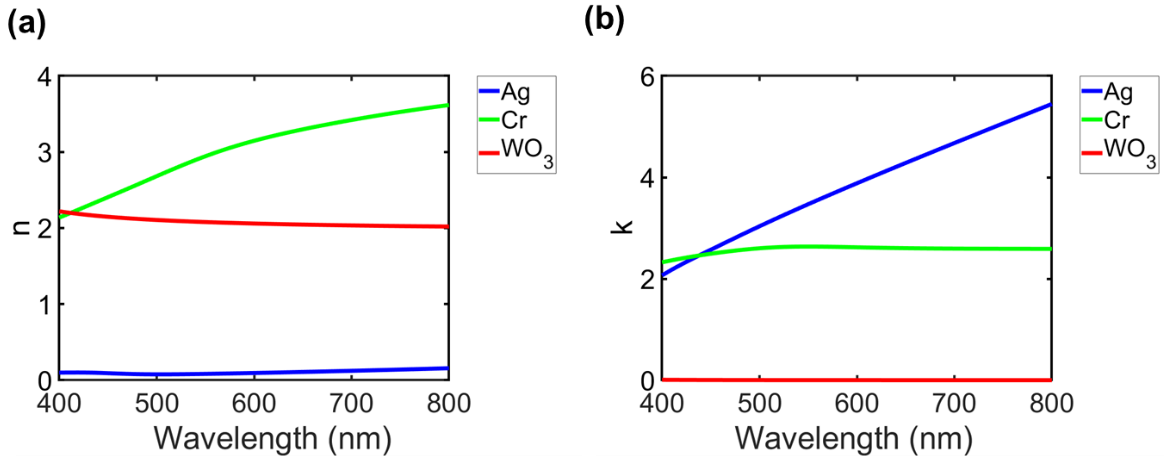

Figure S3. (a) Refractive indices and (b) extinction coefficients of constituent materials.

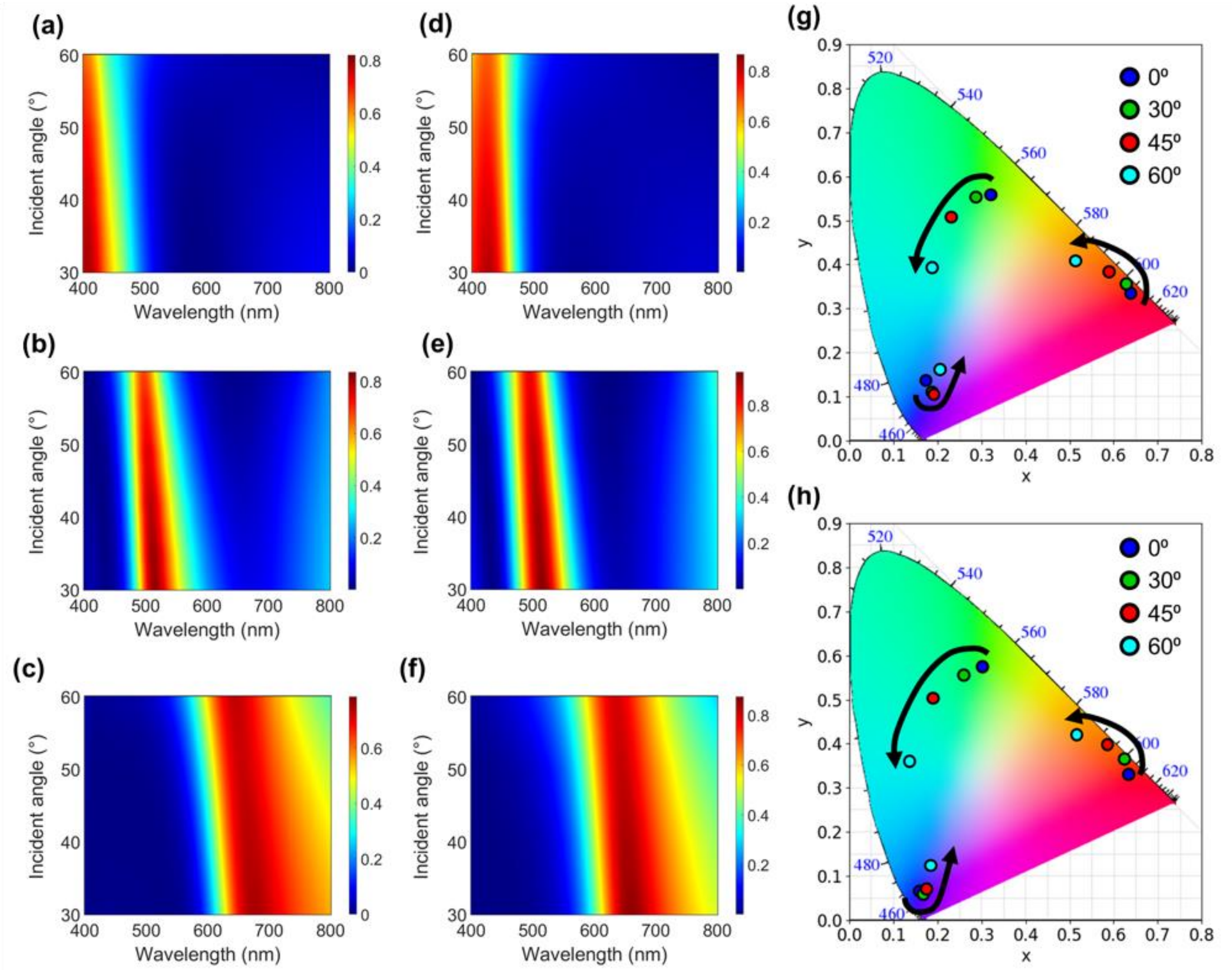

Figure S4. Measured ((a) – (c)) and simulated ((d) - (f)) angle-resolved reflectance spectra of the proposed structural color filters. (g) Measured and (h) simulated color coordinate changes with increasing the incident angles for p-polarization described on CIE 1931 chromaticity diagram.

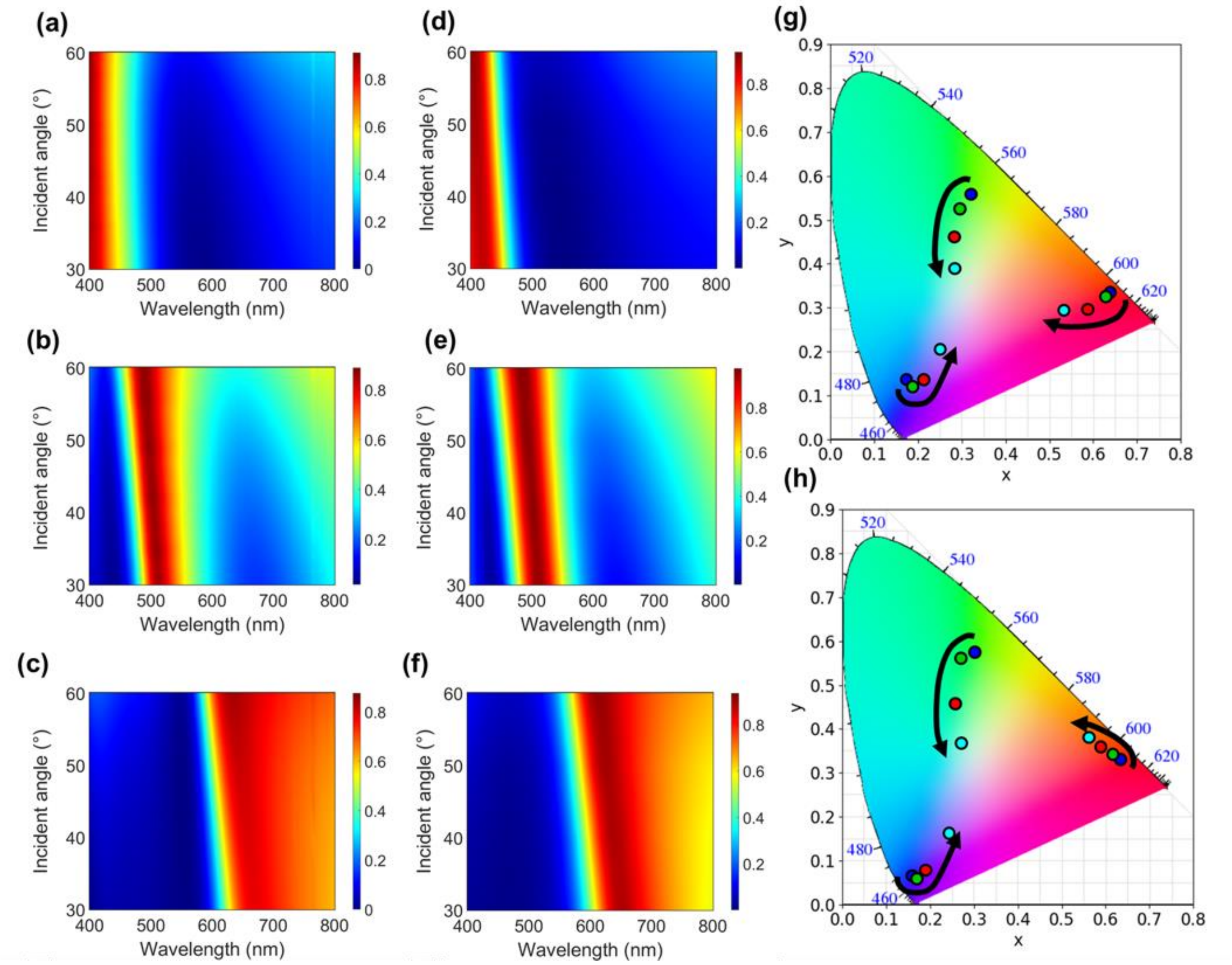

Figure S5. Measured ((a) – (c)) and simulated ((d) - (f)) angle-resolved reflectance spectra of the proposed structural color filters. (g) Measured and (h) simulated color coordinate changes with increasing the incident angles for s-polarization described on CIE 1931 chromaticity diagram.
